# High-throughput single-cell live imaging of photobiomodulation with multispectral near-infrared lasers in cultured T cells

**DOI:** 10.1117/1.JBO.25.3.036003

**Published:** 2020-03-19

**Authors:** Wataru Katagiri, GeonHui Lee, Akira Tanushi, Kosuke Tsukada, Hak Soo Choi, Satoshi Kashiwagi

**Affiliations:** a Massachusetts General Hospital, Gordon Center for Medical Imaging, Department of Radiology, Charlestown, Massachusetts, United States; b Keio University, Graduate School of Science and Technology, Yokohama, Kanagawa, Japan; c Korea University, KU-KIST Graduate School of Converging Science and Technology, Seoul, Republic of Korea; d Massachusetts Institute of Technology, Department of Chemistry, Cambridge, Massachusetts, United States

**Keywords:** photobiomodulation, reactive oxygen species, single-cell live imaging, calcium signaling, near-infrared laser

## Abstract

**Significance:** Photobiomodulation is a well-established therapeutic modality. However, the mechanism of action is poorly understood, due to lack of research in the causal relationship between the near-infrared (NIR) light irradiation and its specific biological effects, hindering broader applications of this technology.

**Aim**: Since biological chromophores typically show several absorption peaks, we determined whether specific effects of photobiomodulation are induced with a combination of two wavelengths at a certain range of irradiance only, rather than a single wavelength of NIR light.

**Approach**: In order to analyze a wide array of combinations of multispectral NIR light at various irradiances efficiently, we developed a new optical platform equipped with two distinct wavelengths of NIR lasers by high-throughput multiple dosing for single-cell live imaging. Two wavelengths of 1064 and 1270 nm were selected based on their photobiomodulatory effects reported in the literature.

**Results**: A specific combination of wavelengths at low irradiances (250 to $400\;\mathrm{mW}/{\mathrm{cm}}^{2}$ for 1064 nm and 55 to $65\;\mathrm{mW}/{\mathrm{cm}}^{2}$ for 1270 nm) modulates mitochondrial retrograde signaling, including intracellular calcium and reactive oxygen species in T cells. The time-dependent density functional theory computation of binding of nitric oxide (NO) to cytochrome *c* oxidase indicates that the illumination with NIR light could result in the NO release, which might be involved in these changes.

**Conclusions**: This optical platform is a powerful tool to study causal relationship between a specific parameter of NIR light and its biological effects. Such a platform is useful for a further mechanistic study on not only photobiomodulation but also other modalities in photomedicine.

## 1 Introduction

Near-infrared (NIR) light (650 to 1700 nm) has been extensively used in various medical procedures and bioimaging with its unique physical properties. NIR light is featured with reduced scattering, minimal tissue absorption with high tissue penetration depth, and low tissue autofluorescence interference compared with visible light, and therefore suitable for bioimaging with high signal-to-background ratio and resolution of deep tissues.^1^^–^^5^ In addition, it shows minimum genotoxicity.^6^ These characteristics collectively favor the therapeutic use of NIR light in the clinic. In fact, since low power NIR light, typically 1 mW to $5\;W/{\mathrm{cm}}^{2}$, shows diverse biological effects, including pain relief, facilitation of tissue regeneration, and reduction of the inflammation,^7^^–^^11^ which is broadly defined as photobiomodulation, the use of NIR light has been explored for a wide array of therapeutic purposes during the past decades.^10^

These beneficial effects of NIR light are mediated by mitochondrial retrograde signaling, including reactive oxygen species (ROS).^7^^–^^15^ Cytochrome *c* oxidase (COX) in electron transport chain (ETC) in mitochondria has been regarded as the primary source of mitochondrial ROS across organisms,^14^ while ROS can be generated across complexes I to IV in the ETC and other compartments in mitochondria.^7^^,^^8^^,^^16^^–^^18^ NIR light has been shown to alter cell metabolism resulting in the generation of ROS^14^^,^^15^ and activation of ROS-mediated retrograde signaling via the class O forkhead box transcription factors (FOXOs), nuclear factor kappa-light-chain-enhancer of activated B cells (NF-κB), activator protein 1 (AP-1), and Myc.^14^ Thus, mitochondrial ETC is regarded as a primary photoreceptor for NIR light.^15^^,^^19^ Interestingly, recent studies demonstrated that a brief exposure of the skin with NIR laser ranging from 1061 to 1301 nm at an irradiance of 0.5 to $5\;W/{\mathrm{cm}}^{2}$ augmented the immune response to intradermal vaccination and conferred protection.^20^^–^^25^ In these studies, the activation of innate responses, subsequent migration of skin-resident dendritic cells, and augmentation of adaptive response were dependent on the generation of ROS.^21^ It has also been reported that broadband NIR light (760 to 1440 nm) induces the formation of mitochondria-derived ROS and subsequent increase in redox potential in cultured human dermal fibroblasts.^26^ Furthermore, 1064-nm NIR light has been shown to modify the function of COX and improve tissue oxygenation in humans.^27^ Although NIR light generally increases the generation of ROS, 950-nm NIR light has been reported to decrease mitochondrial membrane potential and ROS production,^28^ suggesting that photobiomodulation could be dependent on the types of cells, activation status of cells, laser parameters, including wavelengths, irradiance, treatment time, and pulsations. However, there is a paucity of studies to investigate interactions of NIR light of 1000 to 1400 nm with mitochondrial retrograde signaling to advance our understanding on the mode of action of this particular range of NIR light.

The effects of NIR light are also reported to be mediated by calcium signaling, which consists of a significant part of mitochondrial retrograde signaling.^7^^–^^15^ Calcium has multiple important roles in regulating the cellular functions, including survival, death, locomotion, secretion, metabolism, and gene expression.^29^ Light (633 to 980 nm) has been largely reported to increase intracellular calcium,^30^^,^^31^ while 810-nm NIR light could normalize (decrease) the excessively high level of intracellular calcium in a pathological condition,^32^ and 632.8^33^ or 780 nm^34^ light was reported to inhibit calcium uptake by mitochondria depending on an irradiance. Although the precise molecular mechanisms of action for this effect remain unclear, heat- or light-sensitive ion channels have been suggested to be involved in the modulation of calcium signaling in photobiomodulation.^31^ In particular, UV-IR light was reported to modulate the function of transient receptor potential (TRP) channels, including transient receptor potential cation channel subfamily V member 1 (TRPV1), TRPV2, and TRPV4.^31^^,^^35^^–^^39^

Anti-inflammatory effects including pain relief and reduction of the inflammation are a hallmark of photobiomodulation.^7^^–^^11^ For example, this modality can reduce edema, leukocyte infiltration, and the expression of proinflammatory cytokines, accelerating tissue repair of muscles in young individuals.^40^ Accordingly, photobiomodulation on immune cells including macrophages^41^ and dendritic cells^42^ has been reported. Importantly, recent studies consistently show that ROS play a critical role in regulating T cell functions, including T cell receptor (TCR) signaling, T cell proliferation, effector function, and resolution of effector function (death).^43^^,^^44^ ROS are constantly generated in mitochondrial oxidative phosphorylation via electron leak from ETC in T cells.^45^ In particular, mitochondrial ROS has been shown to act on NF-κB and stimulate production of interleukin 2 (IL-2) and other pro-proliferative genes to combat exogenous pathogens or endogenous tumors.^43^ On the other hand, it has been shown that ROS can upregulate FasL and induce T cell death^46^ and that prolonged ROS signaling can suppress T cell responses.^47^ Thus, maintenance of the adequate level of ROS with antioxidant systems within the cell is critical for preserving the integrity of T cell immunity. Similarly, cytosolic and organellar calcium concentrations are well known to control effector functions of T cells. Calcium influx is mediated through a diverse array of receptor- or voltage-activated calcium channels in T cells.^48^^–^^50^ Intracellular organelles, such as the endoplasmic reticulum, mitochondria, and lysosomes, also express specific channels and transporters that contribute to calcium increase in the cytosol and uptake into these organelles.^50^^–^^52^ The strength and duration of calcium signaling have been shown to activate different transcription programs in a T cell subset-dependent manner and determine unique functions, such as metabolism, proliferation, death, differentiation, cytokine secretion, and cytotoxicity of each subset of T cells.^50^ The calcium and ROS signaling is tightly connected to each other in T cells. For example, upon T cell activation, calcium enters the mitochondria and activates enzymes in the TCA cycle, increasing their production of ROS.^53^ Calcium signaling-induced ROS generation has also been shown to play a role in IL-2 production in activated T cells. Recently, it has been demonstrated that defects in mitochondrial complex III cause decreased IL-2 production because ROS regulate the calcineurin nuclear factor of activated T-cells (NFAT) signaling pathway.^44^ As described already, ROS and calcium signaling are critical for both T cell biology and mitochondrial retrograde signaling activated upon photobiomodulation,^9^^,^^11^ we chose a cultured T cell system to establish imaging methodology to examine responses of cells to NIR light for the current study.

Here, we report a high-throughput, single-cell resolution imaging methodology to analyze the effects of multispectral NIR wavelengths at a range of irradiance on intracellular signaling pathways in T cells using established fluorescence imaging *in vitro*.

## 2 Dual-Laser Illumination and Real-Time Single-Cell Live Imaging System

All animal procedures were approved by the Massachusetts General Hospital (MGH) IACUC (protocol number 2009N000103) and performed under the Public Health Service Policy on Human Care of Laboratory Animals.

### 2.1 Optical Setup

**Fig. 1:**
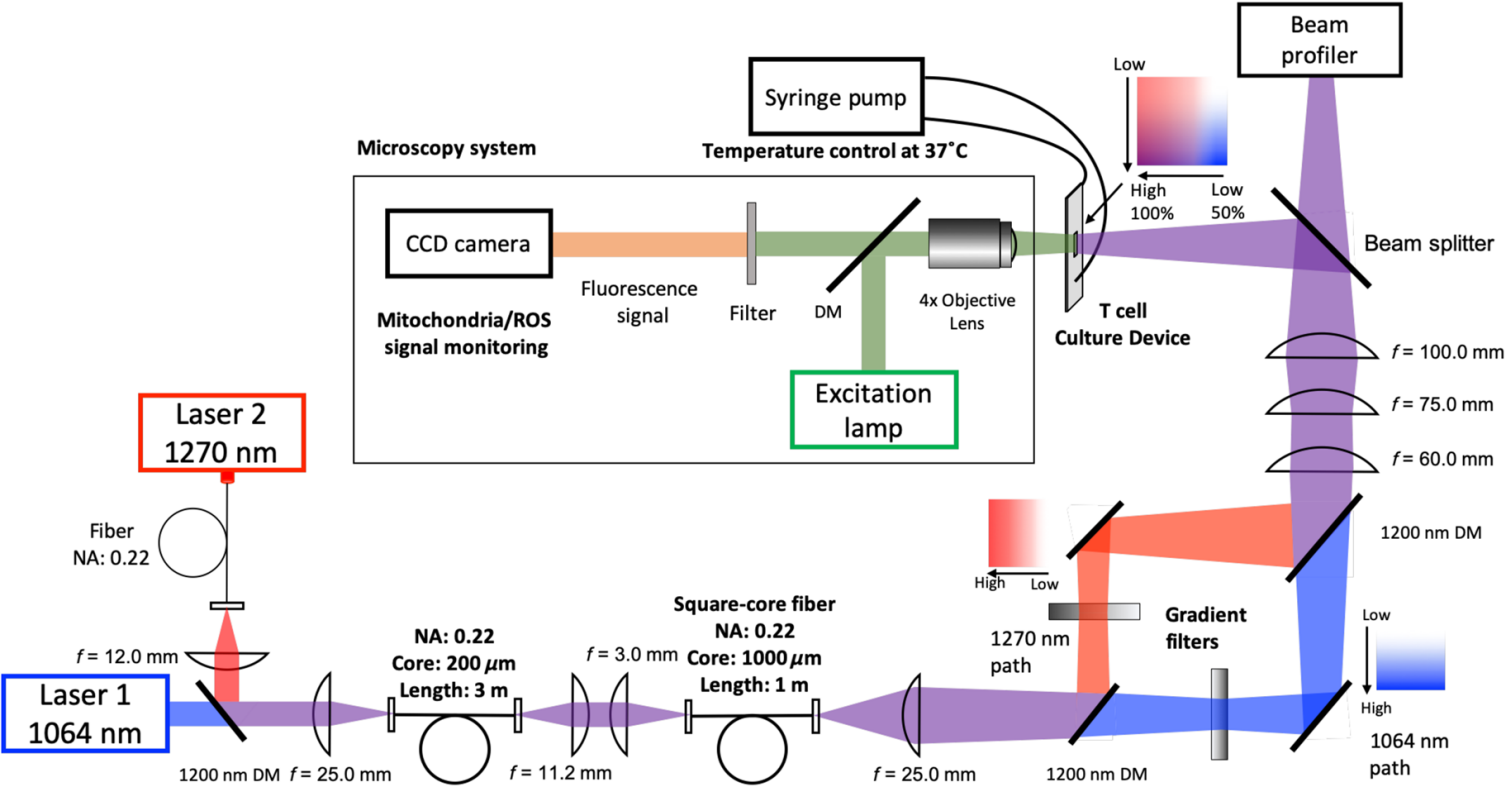
A schematic of the real-time, single-cell live imaging system capable of dual NIR laser irradiation. The 1064- and 1270-nm top-hat square laser beams were transformed into a gradient square beam profile at a focal plane on a cell culture device. The 1064- and 1270-nm laser paths were divided into two through 1200-nm short-pass dichroic mirrors (DMs) to create gradient beams in irradiance and then merged into one square beam. To minimize chromatic aberration, achromatic lenses were used as appropriate in the optical paths. For live imaging, a mercury lamp was used for an excitation light source. The fluorescence signal from cultured cells in the culture chamber was captured by a CCD camera on a fluorescence microscope.

A schematic of the optical system is described in Fig. 1. Continuous-wave Nd:YAG laser (λ=1064  nm, Ventus, Laser Quantum, United Kingdom) and indium phosphide semiconductor diode laser (λ=1270  nm, spectral width=8  nm/3  dB, 202-000, Veralase LLC) were used as sources of NIR light. To distribute the mode equally, these two optic paths were unified and directed through a multimode optic fiber (core=200  μm, NA=0.22, Thorlabs) and 1200-nm short-pass dichroic mirrors (Thorlabs). Then, the beam shape and the homogeneity were adjusted to a square through a vibrating square-core fiber (core=1000  μm, NA=0.22, Fiberguide Industries) at a focal plane. The squared 1064- and 1270-nm laser beams were further divided into two pathways using a dichroic mirror, followed by two-dimensional gradation creation through continuously variable neutral density filters (optical density = 0.04 to 2.00, Thorlabs). Beam shapes of the laser at a desired plane were determined by a beam profiler (Thorlabs Beam 7.0, Thorlabs).

### 2.2 Fabrication of Cell Culture Device for Laser Illumination

**Fig. 2:**
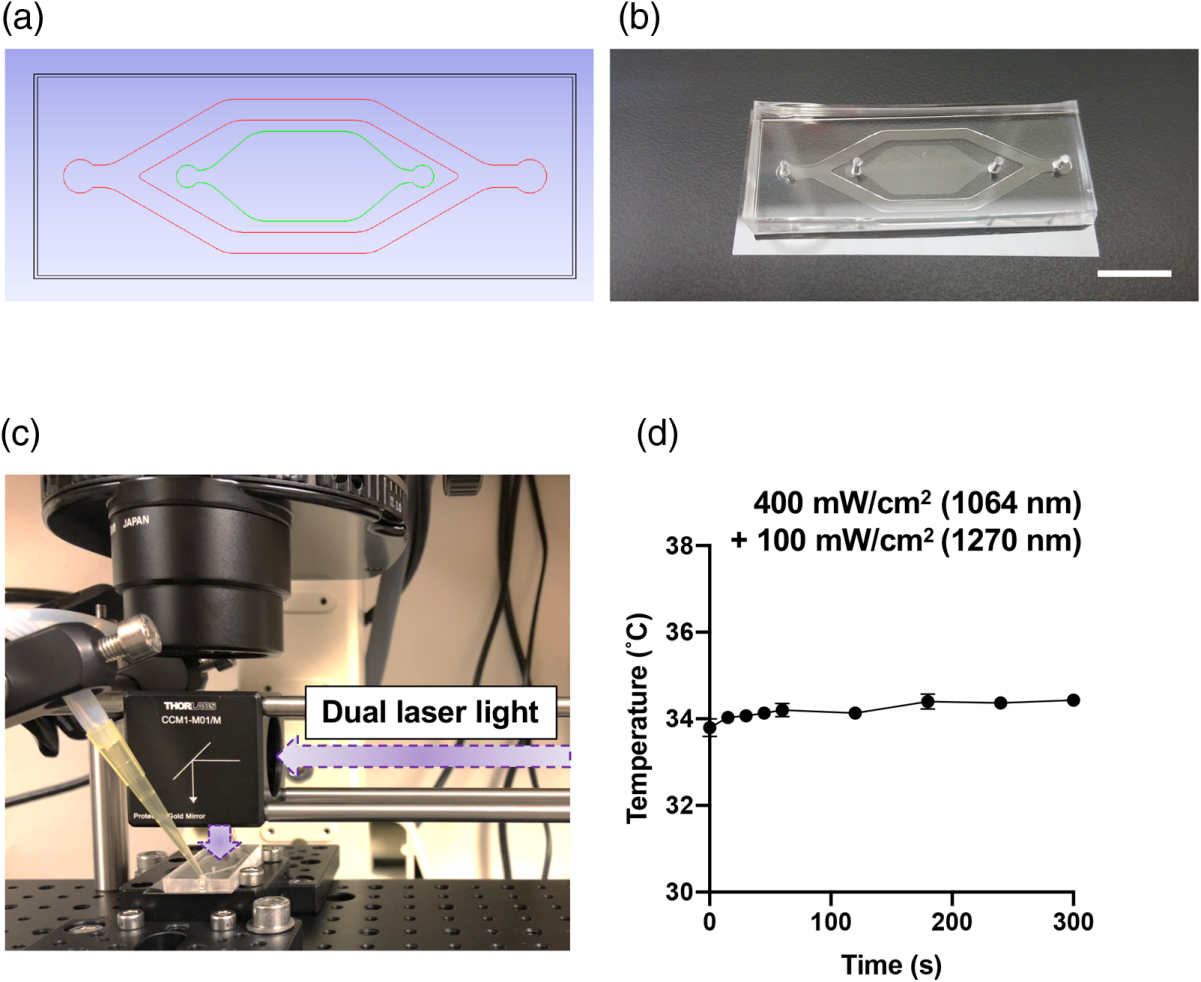
The design of a cell culture chamber for laser irradiation. (a) The design of the PDMS device. Red lines denote a water flow channel for temperature control, and green lines show a cell culture channel. T cells were inoculated and cultured in the cell culture channel without flow. (b) A photo of the PDMS device. Bar=1.0  cm (c) The PDMS device installed on the imaging system. The photo shows a relative location of the laser beam to the chamber with the water flow channel connected to the water circulation system. (d) Measurements of temperature of the culture chamber during dual laser irradiation (n=3). The temperature of the chamber was measured using an IR camera.

The design of the cell culture device is depicted in Figs. 2(a) and 2(b). To achieve single-layer cell culture of T cells with no convection at a constant temperature during laser irradiation, we established a cell culture device using conventional soft lithography. The Si master mold was fabricated using two layers of photoresist. The first layer of photoresist, SU-8 10 (Microchem), for a cell culture chamber was spin-coated at 1250 to 2200 rpm for 40 s (15 to 25  μm). The second layer of photoresist, SU-8 100, for temperature control was then spin-coated at 1100 rpm for 40 s (250  μm). After the development, the resultant wafer was placed in a square dish, and then polydimethylsiloxane (PDMS) prepolymer (a mixture of 10:1 silicon elastomer and curing agent, Sylgard 184, Dow Corning) was poured into it. Then, the square dish was cured in an oven at 70°C for 8 h. In prior to being used, the devices were sterilized by ethylene oxide gas. The water flow channel was connected to a peristaltic pump so that prewarmed water at 37°C flowed into the channel to minimize temperature change of cultured cells in the device, as shown in Figs. 2(c) and 2(d).

### 2.3 T-Cell Culture

Eight-week-old female C57BL/6J mice (stock 000664) were purchased from Jackson Laboratories and acclimated for at least 2 weeks at MGH. The spleen was removed, individually dissociated, and passed through 70- and 40-μm mesh filters to obtain single-cell suspensions of purified T cells. Erythrocytes were then removed using erythrocyte lysing buffer (eBioscience). Splenocytes were further purified to obtain T cells using magnetic beads (EasySep™ T cell isolation kit, STEMCELL Technologies, Canada). The purified T cells were washed and resuspended at a concentration of $1\times 10^{6}\text{ cells}/\mathrm{mL}$ and incubated overnight in RPMI1640 (Thermo Fisher Scientific) containing 10% fetal bovine serum (FBS; Thermo Fisher Scientific), 100  U/mL penicillin/streptomycin (Thermo Fisher Scientific), 0.1% 2-mercaptoethanol (Thermo Fisher Scientific), and 10 mM 4-(2-hydroxyethyl)-1-piperazineethanesulfonic acid (HEPES) buffer (Thermo Fisher Scientific) in a 5% ${\mathrm{CO}}_{2}$ incubator at 37°C.

### 2.4 Laser Irradiation on T Cells and Imaging of Intracellular Calcium Level and ROS Generation

To examine intracellular signaling pathways activated by NIR laser light, we used well-established fluorophores to measure the levels of intracellular calcium and mitochondrial ROS. Purified T cells were loaded with 4  μM Fluo-4 AM (Thermo Fisher Scientific)^54^ and 5  μM MitoSOX Red (Thermo Fisher Scientific)^55^ for 30 min in RPMI1640. The cells were then washed with hank’s balanced salt solution (HBSS) buffer containing 0.5% FBS and 10 mM HEPES. Resuspended cells in 2  μL at a concentration of $8\times 10^{5}\text{ cells}/\mu L$ were put into a cell culture channel on the PDMS device.

The cells on the cell culture channel were irradiated with the dual-squared laser beam for 1 min, as depicted in Fig. 2(c). The gradient irradiance of the two lasers was adjusted from 200 to $400\;\mathrm{mW}/{\mathrm{cm}}^{2}$ for 1064 nm and 50 to $100\;\mathrm{mW}/{\mathrm{cm}}^{2}$ for 1270 nm at the focal plane. The size of a square beam was also adjustable between $1.2\times 1.2\;{\mathrm{mm}}^{2}$ and $4.0\times 4.0\;{\mathrm{mm}}^{2}$. To keep the temperature in the system constant, a peristaltic pump (Cole-Parmer) was used to circulate water warmed up to 37°C into the PDMS device in the water flow channel. The temperature on the cell culture area during dual laser irradiation at an irradiance of $400\;\mathrm{mW}/{\mathrm{cm}}^{2}$ for 1064 nm and $100\;\mathrm{mW}/{\mathrm{cm}}^{2}$ for 1270 nm was monitored using an IR camera (FLIR Systems).

To measure the fluorescence signals, the cells on the PDMS device were illuminated using a mercury lamp (Nikon, Japan). The signals from Fluo-4 (excitation/emission: 494/516  nm) and MitoSOX Red (510/580  nm) were captured using a fluorescence microscopy system (DIAPHOT200, Nikon). The fluorescence signal passed through 4× objective lens (NA=0.13, Nikon) and cube filters were captured by a charge-coupled device (CCD) digital camera (Hamamatsu, Japan). The exposure time of the camera was set to 1000 ms.

### 2.5 Image Analysis

**Fig. 3:**
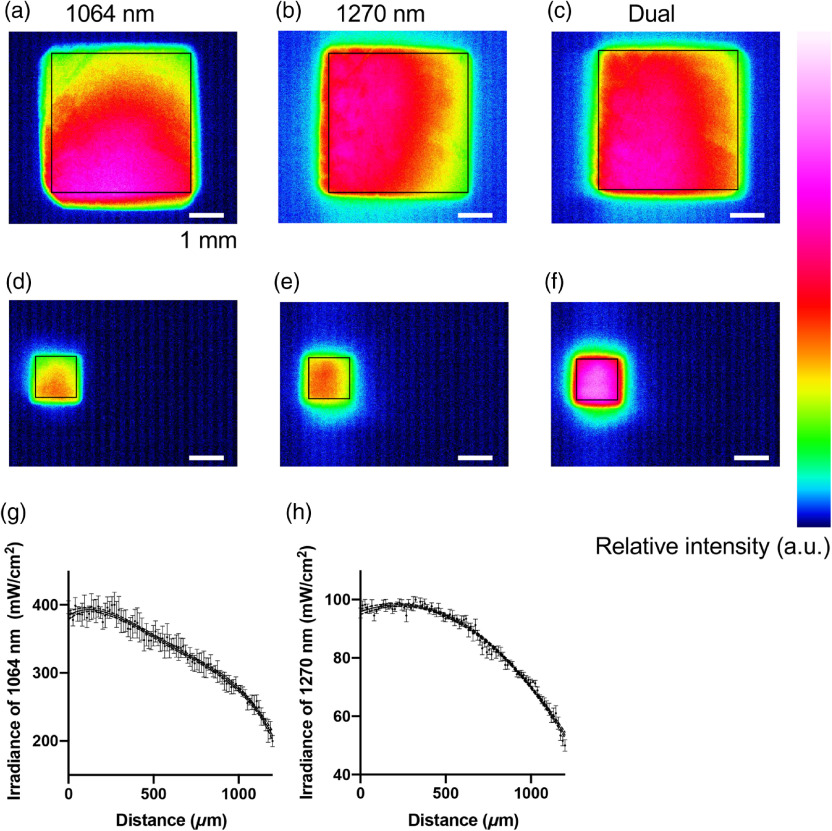
Measurements of the density gradient of the dual laser beam. Relative intensities of different sizes of gradient square beams are shown. The sizes of laser beams are shown in black squares. (a)–(c) $4.0\times 4.0\;{\mathrm{mm}}^{2}$, (d)–(f) $1.2\times 1.2\;{\mathrm{mm}}^{2}$. (a, d) 1064 nm and (b, e) 1270 nm gradient beam were merged into one (c, f). The gradients were fitted into nonlinear curves: (g) 1064 nm [n=5, mean±standard error of the mean (SEM)] and (h) 1270 nm (n=5, mean±SEM). The curve fittings were performed using the least square method, where the degrees of polynomial were selected by the Akaike’s information criterion (AIC). Dash lines show 95% confidence interval (CI).

**Fig. 4:**
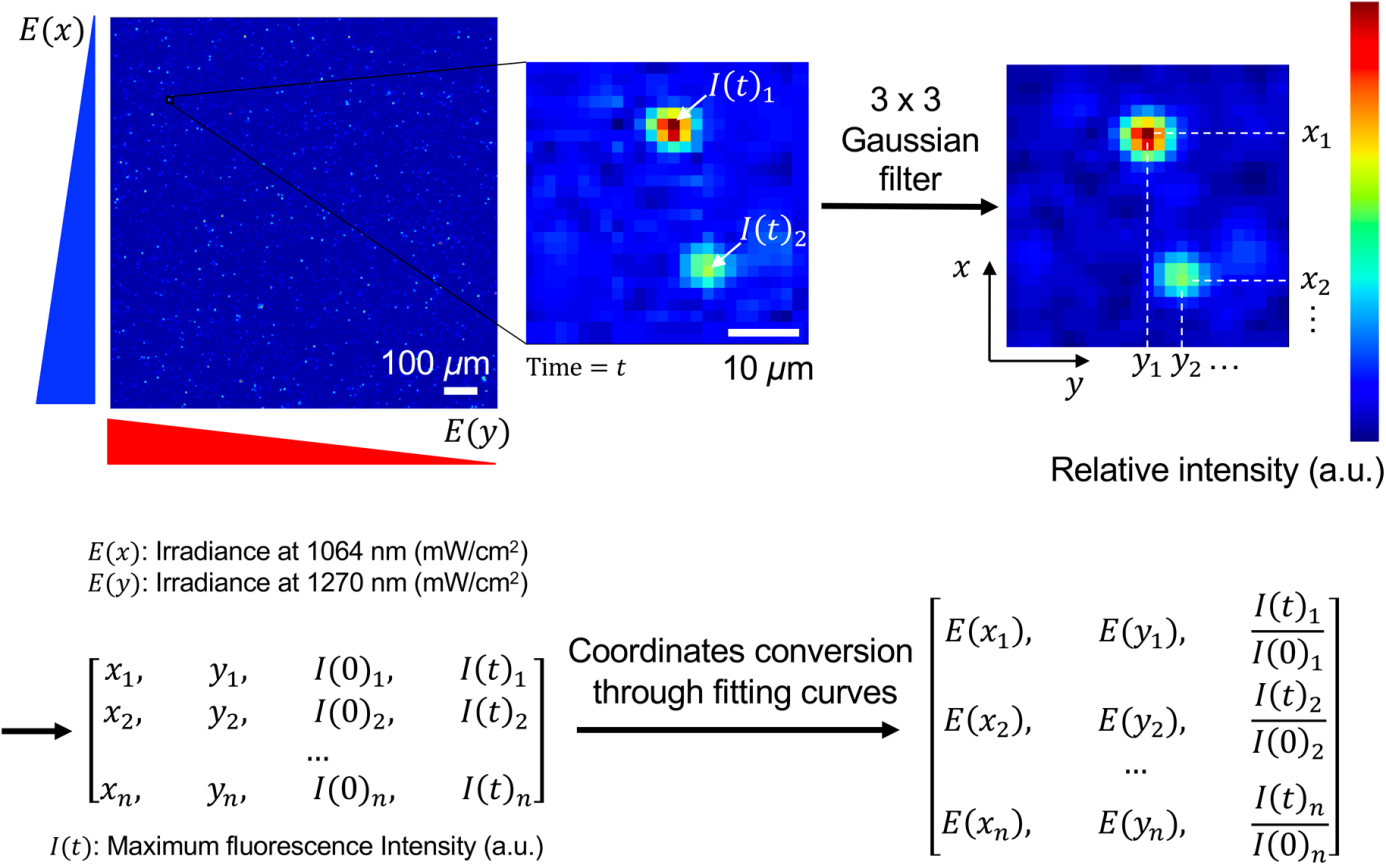
Image analysis strategy. Fluorescence images were captured at 0, 1, and 5 min after the conclusion of laser irradiation. First, the images were convolved with a Gaussian filter. The pixel coordinates with the maximum intensity were defined as a location of individual cells, which were further converted into irradiances of both 1064 and 1270 nm lasers using the curve fittings shown in Figs. 3(g) and 3(h). The fluorescence intensity was measured in the prefiltered images. The fold change of the fluorescence signal at 1 and 5 min over that before laser irradiation was calculated for each time point.

Image analysis scheme is shown in Fig. 4. First, captured images were processed using a 3×3 Gaussian filter to reduce background noise. Second, a pixel, which showed maximum intensity in the nearest 5×5  pixels ($∼8\times 8\;{\mu m}^{2}$) above empirically determined threshold, was recognized as a fluorescence signal. Cells that were stably located at the same position over time were selected for further analysis. A fold change of the fluorescence signal intensity at a desired time point over the signal before the illumination was calculated. The cells’ geometric coordinates were converted into irradiances using curve fittings for the dual square beam, as shown in Fig. 3. The analysis software program was developed using a programming language Python 3.6 and OpenCV 2 (programs are available at https://github.com/wkatagiri/CB001).

### 2.6 Time-Dependent Density Functional Theory Analysis of Nitric Oxide Binding to Cytochrome *c* Oxidase

We used the time-dependent density functional theory (TDDFT) to estimate the absorption spectrum of COX. TDDFT calculations were carried out using Gaussian 09W program package.^56^ The three-parameterized Becke–Lee–Yang–Parr (B3LYP) hybrid exchange-correction functional was employed^57^^–^^59^ and 6-311G** was used as a basis set.^60^^–^^62^ Solvent effects were not considered in any of the processes.

**Fig. 6:**
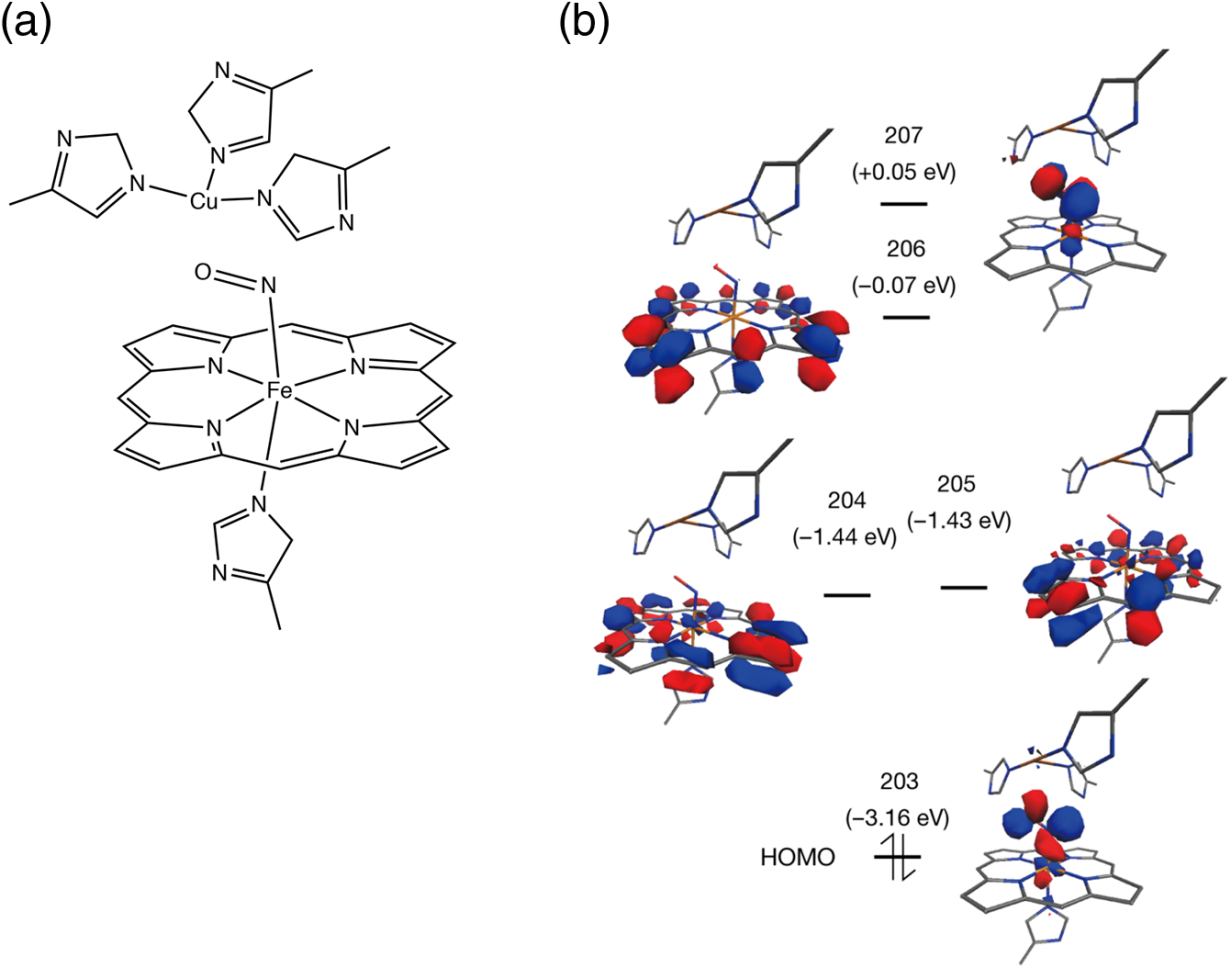
TDDF analysis. (a) A part of molecular structure of COX (PDB: 5X1F) (structure 1). Approximately, a region in a diameter of 8 Å from NO molecule was extracted from COX. A copper ion is surrounded by three histidines, whereas NO binds to an iron ion, which is coupled with porphyrin structure and a histidine. (b) Calculated MOs for complex 1 at B3LYP/6-311G** level and the orbital diagram.

A specific region around copper ion–nitric oxide–iron ion (Cu-(NO)-Fe) binding with porphyrin complex and histidine (His) was extracted from the whole COX. The model structure is depicted in Fig. 6(a): $3\mathrm{His}\text{-}\mathrm{Cu}\text{-}(\mathrm{NO})\text{-}\mathrm{Fe}(C_{20}N_{4}H_{12})$-His (structure 1). On the basis of the optimized structure 1, the TDDFT method was applied to calculate the excited states relevant to the NIR absorption.

### 2.7 Statistical Analysis

One-way analysis of variance (ANOVA) followed by Tukey’s multiple comparison tests was performed for comparisons of more than two groups using GraphPad Prism version 8. The mean±standard error of the mean was displayed for all figures. A multiple comparison test’s corrected P value <0.05 was considered to be significant.

## 3 Results

### 3.1 Development of Optical Platform Equipped with Two Distinct Wavelengths of NIR Lasers

We first constructed a culture chamber for T cells, which was amenable for laser illumination. We designed the optical platform using computer-aided software [Fig. 2(a)] and developed by conventional photolithography and soft lithography [Fig. 2(b)]. The device is $18\times 48\;{\mathrm{mm}}^{2}$ in size and consists of two channels: a water flow channel (shown in red) and a cell culture channel (green). The water flow channel was connected to tubes to circulate warmed water while the laser was irradiated in the middle of the cell culture area, as shown in Fig. 2(c). The water flow channel was designed to accommodate water flow at a speed of up to 30  mL/min through peristaltic pumping. The cell culture channel was surrounded by the water flow channel to achieve homogeneous temperature distribution over the cell culture area. To assess the thermal transfer from water channel to cell culture chamber, we monitored the temperature distribution of the cell culture area in real-time during laser irradiation measured by an IR camera [Fig. 2(d)]. The temperature was stable between 33°C and 35°C during simultaneous laser irradiation with $400\;\mathrm{mW}/{\mathrm{cm}}^{2}$ of 1064 nm and $100\;\mathrm{mW}/{\mathrm{cm}}^{2}$ of 1270 nm up to 5 min. Hence, the effect of heat generation with laser irradiation on cultured cells in the channel was considered negligible in the following analysis, which used lower irradiances of the NIR laser for up to 1 min than those for the temperature measurement.

### 3.2 Dual Laser Beam Shaping

Next, we constructed an optical system, where we irradiated the cultured cells with NIR laser and performed bioimaging at the same time. Two different sizes of the square beams were created by adjusting positions of planoconvex and achromatic lenses between gradient filters and the cell culture device [Figs. 3(a)–3(f)]. The size of the square beam varied from $1.2\times 1.2\;{\mathrm{mm}}^{2}$ to $4.0\times 4.0\;{\mathrm{mm}}^{2}$. The smallest $1.2\;{\mathrm{mm}}^{2}$ beam was adjusted to the field of view through 4× objective lens (NA=0.13, Nikon), whereas the largest $4.0\;{\mathrm{mm}}^{2}$ beam was adjusted for 2.5× objective (NA=0.075, Nikon). Although both the smallest and largest squares could be applied for illumination sources, intracellular fluorescence signals in T cells could not be clearly recognized by the CCD camera with 2.5× objective lens (data not shown). Therefore, we conducted the following experiments with the smallest $1.2\;{\mathrm{mm}}^{2}$ using the 4× objective lens.

Curve fittings of irradiance gradient of 1064 and 1270 nm in the field are shown in Figs. 3(g) and 3(h). The line profiles of irradiance were measured toward the gradient vectors with a beam profiler. The measurement results were plotted, and fitting curves for each wavelength were then calculated based on the least square method. The gradient of irradiance of 1064 nm was fitted into quartic function E(x) [Eq. (1)], whereas that of 1270 nm was fitted into quadratic function E(y) [Eq. (2)] as shown as follows:

$$E(x)=384.3+(0.1546)x-(7.934\times 10^{-4})x^{2}+(9.238\times 10^{-7})x^{3}-(3.934\times 10^{-10})x^{4},$$ (1)

$$E(y)=95.82+(2.091\times 10^{-2})y-(4.679\times 10^{-5})y^{2}.$$ (2)

The degree of each fitting curve function was determined by Akaike’s information criterion. The narrow 95% confidence interval of each fitting curve, as shown in Figs. 3(g) and 3(h), suggests that the fitting strategy used here was satisfactory and the irradiance gradient of the two lasers was established successfully with the optical setting.

### 3.3 Calcium and ROS Responses upon NIR Laser Irradiation

Both intracellular calcium and mitochondrial ROS signaling play a critical role in regulating diverse T cell functions.^43^^,^^44^^,^^48^^–^^50^ Therefore, we analyzed calcium and mitochondrial ROS responses in T cells induced by NIR laser irradiation using the platform established already.

T cells loaded with either calcium probe Fluo-4 or ROS probe MitoSOX Red in the cell culture channel in the PDMS device were irradiated with the dual-squared laser beam at a gradient irradiance of 200 to $400\;\mathrm{mW}/{\mathrm{cm}}^{2}$ for 1064 nm and 50 to $100\;\mathrm{mW}/{\mathrm{cm}}^{2}$ for 1270 nm laser. The fluorescence images were acquired using a fluorescence microscopy system equipped with a CCD digital camera. A fold change of the fluorescence signal intensity at a desired time point over the signal before the illumination was calculated for individual T cells on the captured images. As shown in Fig. 4, geometric information of each T cell in the imaging field was converted into irradiance of 1064 and 1270 nm using curve fitting with a nonlinear regression method.

**Fig. 5:**
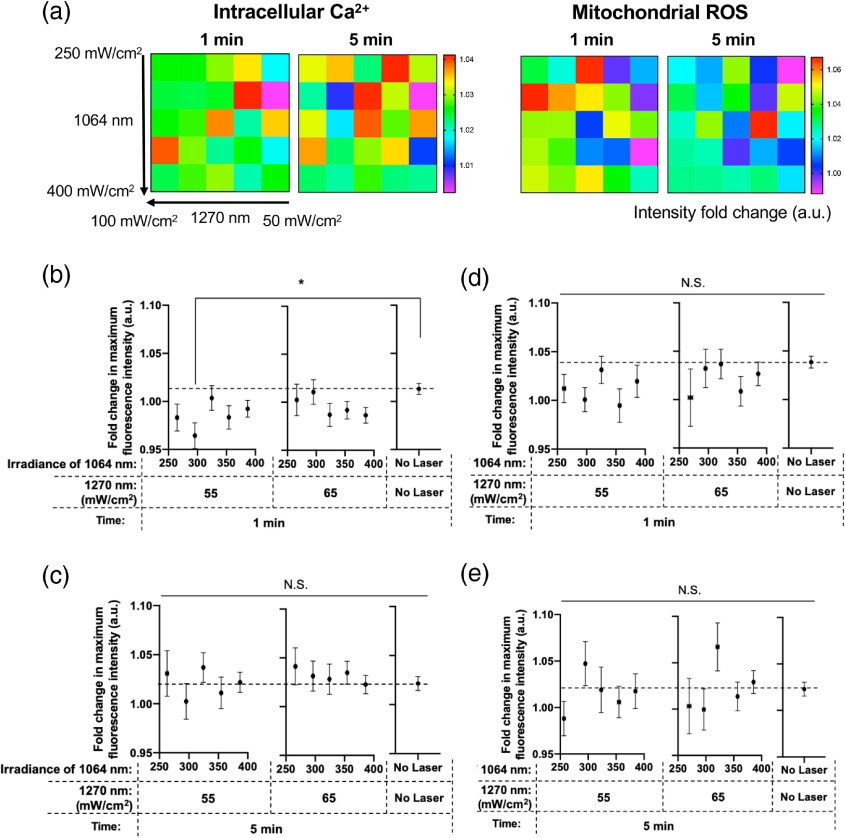
The fold change of the fluorescent signal of intracellular calcium and ROS upon dual NIR laser exposures. The fold change of the fluorescence signals at each time point over the signals before the laser irradiation was calculated for individual T cells on the captured images. (a) Color maps of the fluorescent signal changes 1 and 5 min after the laser irradiation. (b, c) The fold changes of the intracellular calcium signal at (b) 1 min (n=30 to 284) and (c) 5 min (n=20 to 249). (d, e) The fold changes of the ROS signal at (d) 1 min (n=9 to 111) and (e) 5 min (n=7 to 92). (a)–(e) The results under gradient irradiances between 250 and $400\;\mathrm{mW}/{\mathrm{cm}}^{2}$ for 1064 nm and 50 to $100\;\mathrm{mW}/{\mathrm{cm}}^{2}$ for 1270 nm were shown. Results were pooled from two independent experiments. Error bars for x and y axes denote the SEM. A P value <0.05 was considered significant: *P<0.05; N.S., not significant by one-way ANOVA followed by Tukey’s multiple comparison test.

Figure 5(a) shows the color map of fold changes in fluorescence intensity at 1 and 5 min after the laser irradiation. Here, we divided the population of the T cells into 25 groups based on the irradiance of 1064 and 1270 nm exposed to the cells and showed the mean fluorescence intensity of each group. Interestingly, both calcium and mitochondrial ROS signals showed a tendency to decrease (<1.0) after the laser irradiation [Fig. 5(a)]. We then compared the fold changes across combinations of lower irradiances of 1064 and 1270 nm laser [Figs. 5(b)–5(e)]. In these comparisons, the fluorescence signals at 1 and 5 min were compared with those at 0 min (before laser) to take various factors, including photobleaching and leakage of the fluorophore over time into consideration. Notably, there was a clear tendency that combinations of relatively low irradiances of laser with 250 to $400\;\mathrm{mW}/{\mathrm{cm}}^{2}$ for 1064 nm and 55 to $65\;\mathrm{mW}/{\mathrm{cm}}^{2}$ for 1270 nm laser suppressed calcium at 1 min [Fig. 5(b)]. In addition, the calcium signal was significantly decreased with dual irradiation with $300\;\mathrm{mW}/{\mathrm{cm}}^{2}$ of 1064 nm and $55\;\mathrm{mW}/{\mathrm{cm}}^{2}$ of 1270 nm laser compared with that of no laser control group (p=0.0402). The signal went back to the control level 5 min after the laser irradiation [Fig. 5(c)]. Although statistical analysis did not indicate any significant difference, mitochondrial ROS also decreased with irradiances of laser with 250 to $400\;\mathrm{mW}/{\mathrm{cm}}^{2}$ for 1064 nm and 55 to $65\;\mathrm{mW}/{\mathrm{cm}}^{2}$ for 1270 nm laser at 1 min [Fig. 5(d)]. The change in the ROS signal also disappeared 5 min after the laser treatment [Fig 5(e)]. Together, these results indicate that a specific combination of low irradiance at 1064 and 1270 nm lasers suppresses intracellular calcium and mitochondrial ROS signal in T cells.

### 3.4 TDDFT Computation of the Effect of NIR Light on NO Binding to COX

In order to understand the molecular mechanisms of action of these changes in ROS and calcium with NIR light beyond 1000 nm, we performed TDDFT computation of binding of NO to COX molecule (Fig. 6). The results indicate that there are three multispectral absorbance peaks in the NIR range: 961, 1319, and 1372 nm (Table 1). These peaks derive from the electronic transition from molecular orbital (MO) 203 [highest occupied molecular orbital (HOMO)] to low-lying unoccupied orbitals (MOs 204 to 209). Figure 6(b) displays the MOs calculated for structure 1. While the HOMO is centered at the bent Fe–N–O bonds and has the character of backbonding from Fe $d_{\mathrm{xz}}$ orbital to NO π* orbital, the unoccupied orbitals do not have the bonding interaction character between Fe and NO; MOs 204 and 205 are nearly degenerated $e(\mathrm{Fe}\;d_{\pi }-\pi_{\mathrm{por}}*)$ orbitals, MO 206 is centered at the porphyrin ring and MO 207 shows the antibonding interaction between Fe and NO. Therefore, the electronic transitions from HOMO caused by NIR light irradiation are expected to weaken the bonding between the Fe center and NO ligand. The calculated absorption peaks in the NIR spectrum at 961, 1319, and 1372 nm indicate that the illumination with NIR light could result in the NO release in deep thickness of exposed tissue (e.g., the surface of epidermis to the deepest layer of dermis) due to its maximum depth of penetration in biological tissue.^63^

**Table 1:** Predicted absorption of model complex in near-infrared region by TDDFT method at B3LYP/6-311G** level.

$\lambda_{\mathrm{calc}}$^a^/nm	$E_{\mathrm{calc}}$^b^/eV	Oscillator strength^c^	Configuration	Coefficient
1372.32	0.90	0.0001	203 → 204	0.574
			203 → 205	−0.406
1318.91	0.94	0.0004	203 → 204	0.406
			203 → 205	0.574
961.32	1.29	0.0002	203 → 206	−0.102
			203 → 207	−0.682
			203 → 209	0.115

a Absorption maxima.

b Corresponding transition energy calculated by TDDFT method.

c Oscillator strength calculated by TDDFT method.

## 4 Discussion

In the current study, we have established for the first time a high-throughput imaging system at a single-cell resolution to evaluate responses of intracellular calcium and ROS signaling pathways in order to determine an optimal parameter of NIR laser for effective photobiomodulation based on an established fluorescence imaging *in vitro*. Since the effects of NIR light are diverse and the range of the wavelength and irradiance of effective NIR light is wide, seeking for an optimal parameter for a desirable biological effect is often challenging. This platform is, therefore, a powerful tool to identify the best-performing combination of NIR wavelength and irradiance not only for photobiomodulation but more broadly for other applications in photomedicine, including photodynamic and photothermal therapy, contributing to maximizing the efficacy and safety of these treatment modalities.

There are two new features in this optical platform. First, we chose to use PDMS elastomer for an irradiation chamber. PDMS is widely used in biomedical applications owing to its excellent flexibility and transparency in the visible light spectrum.^64^ PDMS also shows very little optical insertion loss in the NIR light spectrum, such as in 900 to 1100 nm and 1250 to 1350 nm to the extent of <0.03  dB/cm.^65^ Therefore, PDMS can be applied for NIR illumination with minimal loss of light intensity and heat generation. Since the temperature during the dual laser irradiation at the large irradiance of $400\;\mathrm{mW}/{\mathrm{cm}}^{2}$ for 1064 nm and $100\;\mathrm{mW}/{\mathrm{cm}}^{2}$ for 1270 nm was measured constant for a long time period, the effect of heat generation upon laser illumination, which might cause intracellular ROS production^66^ and intracellular calcium accumulation,^67^ can be disregarded in the current study. Thus, this platform allows us to determine the effect of photochemical effect of the NIR laser separately from its photothermal effect. Second, the system using a square beam profile allows for a simultaneous high-throughput measurement of photochemical effect of multispectral laser with a wide range of irradiance. Generally, a Gaussian beam profile is used to determine the threshold of monospectral laser in an *in vitro* setting.^68^^–^^70^ However, in the current study, a square gradient beam profile was chosen as appropriate for a dual laser in order to simplify the experimental procedures. We used a square-core fiber to create a square beam profile because the use of the fiber is easier and more affordable than the phase gating^71^ or a micromirror device.^72^ Such a system is useful to study the biological effect of NIR light. Exposures to NIR light between 760 and 1400 nm have been demonstrated to increase ROS generation in a wide variety of cells and tissues, including tumor cells,^73^^–^^75^ keratinocytes,^74^ fibroblasts,^26^ the skin tissue.^76^^–^^78^ ROS have been postulated to act on mitochondrial COX in ECT in response to NIR light exposure,^16^^,^^79^ ultimately resulting in photobiomodulation.^7^^–^^9^ These studies generally used broadband light, which renders interpretation of the impact of such light on the biological system difficult. Since theoretical^80^ and *in vitro* studies^81^^,^^82^ showed that there are absorbance peaks of coppers in the putative photoreceptor COX in the NIR spectra at 620, 680, 760, and 820 nm, biological readouts upon photobiomodulation could be a result of significant effects of one distinct or a combination of several wavelengths of NIR light. In order to further understand the mechanism of action of photobiomodulation, it is desirable to establish a reproducible tool to examine the effect of individual or a combination of parameters of NIR light on physiological functions. In addition, a wide range of power of NIR light (1 mW to $5\;W/{\mathrm{cm}}^{2}$) has been reported to promote photobiomodulation,^7^^–^^11^ whereas over-dosed photoirradiation has been reported to downregulate the signaling pathways,^8^^,^^83^^,^^84^ such a tool should be able to examine a reasonably wide range of parameters simultaneously to identify a causal NIR laser parameter. In response to this, the current system was designed to have the capability to evaluate a combination of multiple wavelengths and a wide range of irradiance simultaneously.

Modulation of interactions between COX and NO in ETC has been linked to ROS generation.^85^ NO is generated by mitochondrial NO synthase in the mitochondrial inner membrane.^86^^,^^87^ NO is able to bind to the oxygen-binding site in competition with oxygen and act as a reversible inhibitor of COX at low concentrations, diverting the fate of oxygen into ROS formation.^88^^–^^91^ NIR light has been shown to alter cell metabolism and induce ROS generation as it displaces NO molecules on histidine in COX subunit I between copper and iron.^88^^–^^91^ While most of the studies on photobiomodulation employed visible and NIR light below 950 nm,^18^^,^^83^^,^^92^^,^^93^ some of the recent studies indicate modulatory effect of NIR light beyond 1000 nm on COX. Wang et al. demonstrated that a brief exposure of the skin of human subjects with 1064-nm NIR light at an irradiance of $250\;\mathrm{mW}/{\mathrm{cm}}^{2}$ induced COX and hemoglobin oxygenation.^27^ In contrast, Sanderson et al. showed that COX activity was strongly suppressed by dual irradiation with 750- and 950-nm lasers.^28^^,^^94^ Consistently, the results of TDDFT computation of the binding of NO to COX molecule indicate that there are three multispectral absorbance peaks in the NIR range (Fig. 6 and Table 1). We further demonstrated that a combination of 1064 and 1270 nm laser suppressed mitochondrial retrograde signaling, including intracellular calcium and mitochondrial ROS (Fig. 5). These results are consistent with our previous study, showing that 1064-nm NIR laser-induced ROS generation in skin cells.^21^ Furthermore, Schroeder et al. similarly demonstrated that broadband NIR light (760 to 1440 nm) induced mitochondria-derived ROS generation and subsequent increase in redox potential in cultured human dermal fibroblasts.^26^ Together with our previous studies showing the immunomodulatory effect of NIR light ranging from 1061 to 1301 nm^20^^–^^23^ and reports that much higher dose of NIR laser light would be needed for direct production of singlet oxygen,^95^ these results suggest that NIR light beyond 1000 nm shows photobiomodulation via mitochondrial retrograde signaling and that the mechanisms of action of photobiomodulation with NIR light beyond 1000 nm might involve dissociation of NO from COX.

ROS are known to regulate the differentiation and effector functions of T cells. High environmental ROS favor the development of T helper ($T_{H}$) two cells with increased IL-2 and IL-4 production, skewing toward a $T_{H}2$-skewed immune response.^96^^,^^97^ In contrast, low ROS rather promote $T_{H}1$ and $T_{H}17$ cell differentiation, and the use of antioxidants increases interferon-γ production, skewing the immune response to a $T_{H}1$ phenotype.^96^^,^^98^^,^^99^ As such, this system can be useful for discovery research to identify a laser parameter modulating T cell function for therapeutic purposes. For example, with this system, we could identify a laser parameter to potentially ameliorate autoimmune inflammation in multiple sclerosis (MS). MS is a neurodegenerative disorder and characterized by the infiltration of autoreactive CD4 T cells against myelin into the central nervous system (CNS).^100^ Since the autoreactive T cells are known to secrete high levels of $T_{H}1$ and $T_{H}17$ cytokines and the administration of pro-oxidants prevents the production of these inflammatory cytokines as high environmental levels of ROS promote the development of $T_{H}2$ cells,^96^^,^^98^ a parameter of NIR light to modulate ROS in T cells may reduce the severity of MS. In fact, a recent report shows that the modulation of the Nrf2-mediated antioxidant pathway in T cells prevents CD4 T cell infiltration into the CNS, thereby ameliorating autoimmune inflammation,^101^ suggesting that this approach would have a clinical impact. In addition, this system could also be used to identify a laser parameter to augment antitumor response. It is well established that T cells play a critical role in antitumor immunity. Cancer is known to produce large amounts of ROS, which suppress immune function of T cells. Consistently, cellular antioxidant levels have been demonstrated to be critical for the antitumor function of T cells in the immunosuppressive tumor microenvironment. Recent study has shown that central memory T cells with higher cytosolic glutathione, surface thiol, and intracellular antioxidant levels survive longer in tumor and control tumor growth than effector memory T cells with less antioxidant levels and that treatment with antioxidants improved the function of tumor-infiltrating T lymphocytes (TILs), leading to prolonged survival of patients receiving these treated TIL.^102^ Further work is warranted to determine if this system could be broadly used to discover such a combination parameter of NIR lasers.

The research investigation on the mechanism of action of photobiomodulation has been constantly evolving for the past two decades. Accordingly, recent studies show that alternative pathway(s) could exist for photobiomodulation other than ROS and calcium, including direct cell-free light-mediated effects on transforming growth factor beta (TGF-β) or adenosine triphosphate (ATP), activation of signaling pathways, including hypoxia-inducible factor 1-alpha (HIF-1α) Akt/glycogen synthase kinase-3 beta (GSK3β)/β-catenin, extracellular signal-regulated kinases (ERK)/forkhead box protein M1 (FOXM1), peroxisome proliferator-activated receptor gamma (PPARγ), runt-related transcription factor 2 (RUNX2), or direct production of the singlet oxygen.^31^^,^^36^^,^^73^^–^^75^^,^^103^^,^^104^ This imaging system could be used to further examine the involvement of these possible pathways in photobiomodulation.

## 5 Conclusion

We have established an optical platform, which allows for real-time, single-cell live imaging of the intracellular signaling induced by multiple doses of two wavelengths of NIR laser light simultaneously *in vitro*. Armed with this system, we revealed that 1-min exposure of cultured T cells with a specific combination of 1064 and 1270 nm NIR lasers at low irradiances suppressed intracellular calcium and mitochondrial ROS signal. These results indicate that a parameter of NIR light beyond 1000 nm promotes photobiomodulation. This novel system would be not only useful for further mechanistic study of photobiomodulation but also a powerful tool to investigate optical and biological responses in photomedicine.
